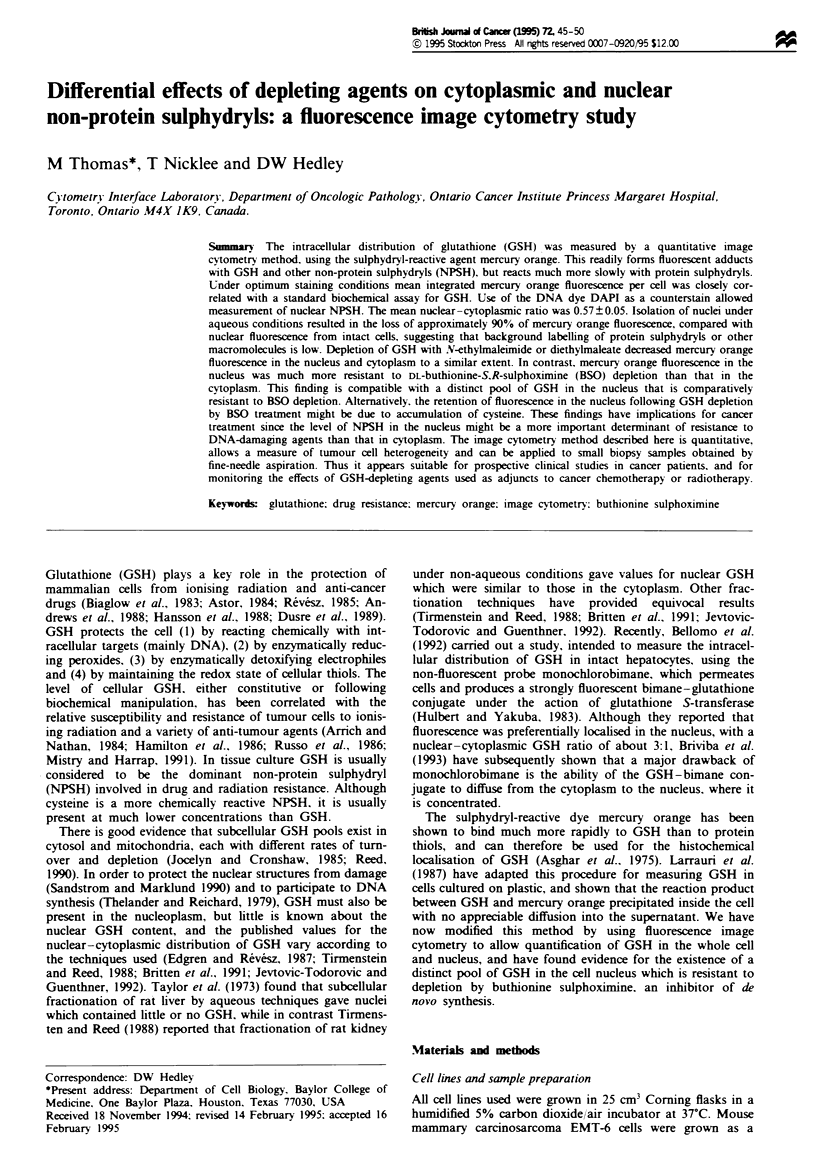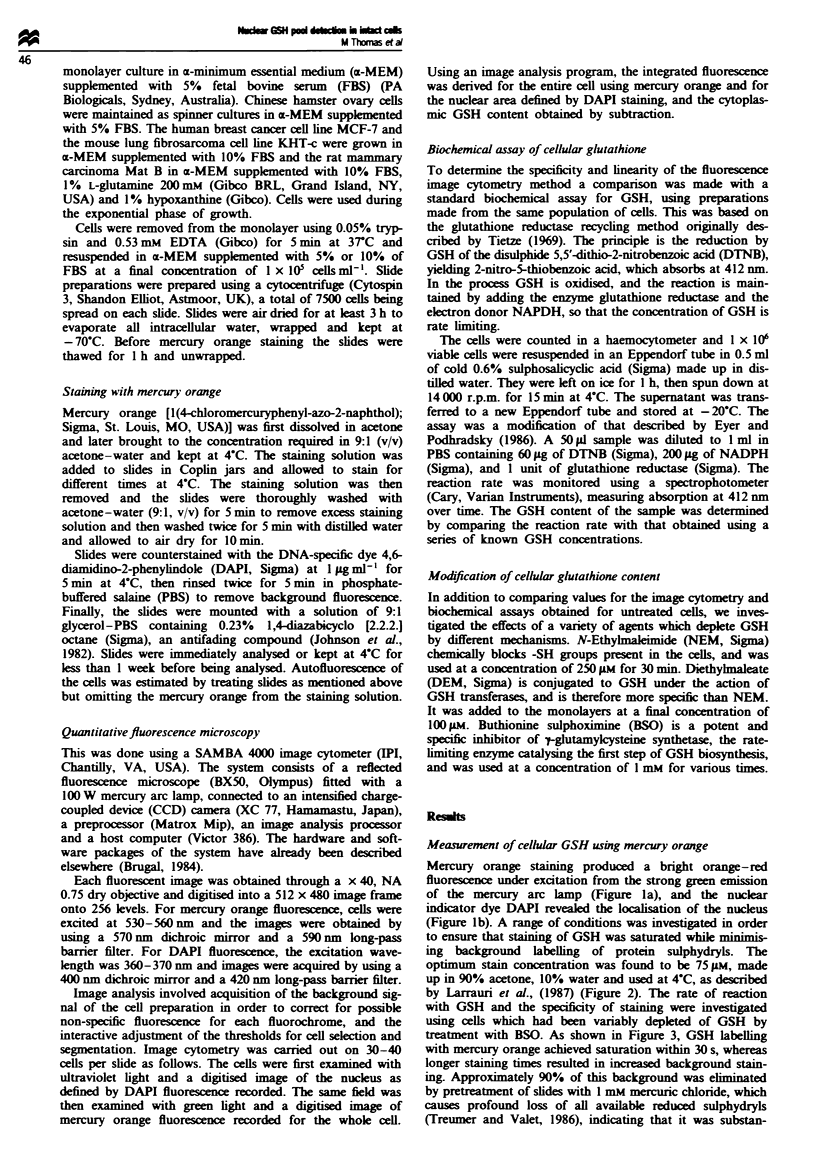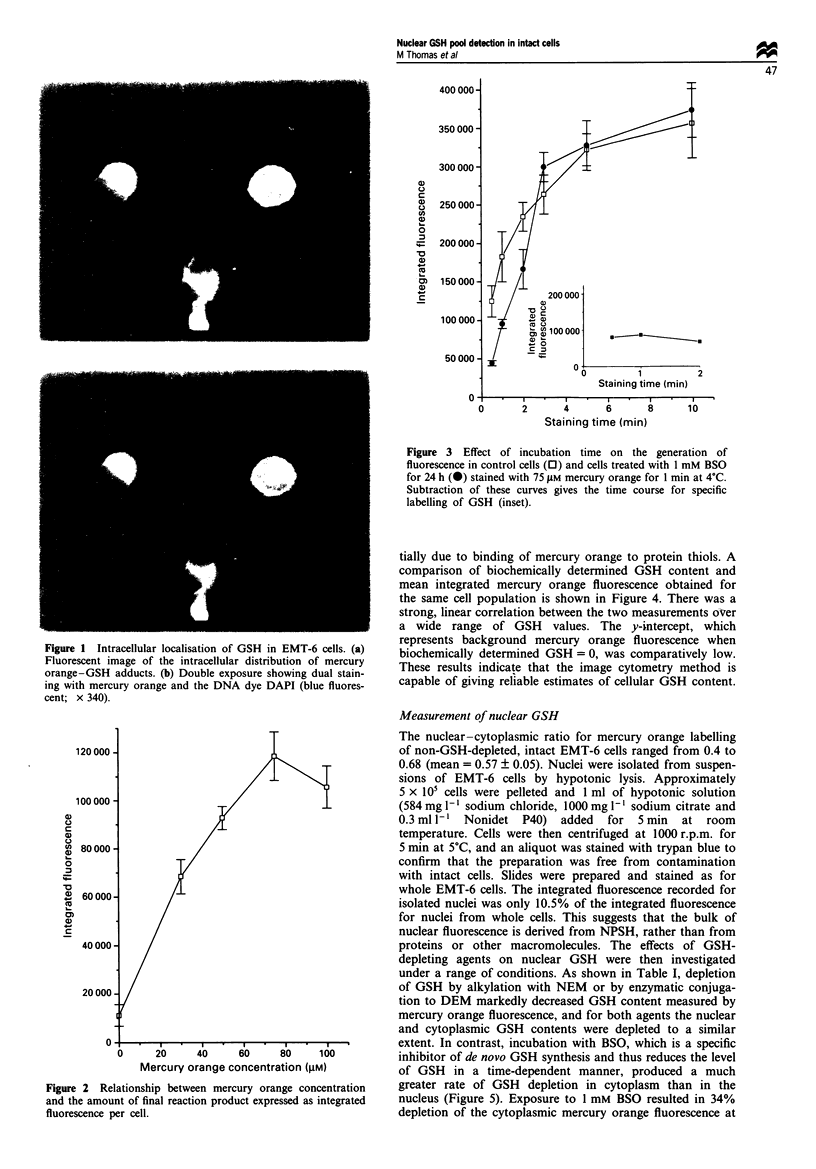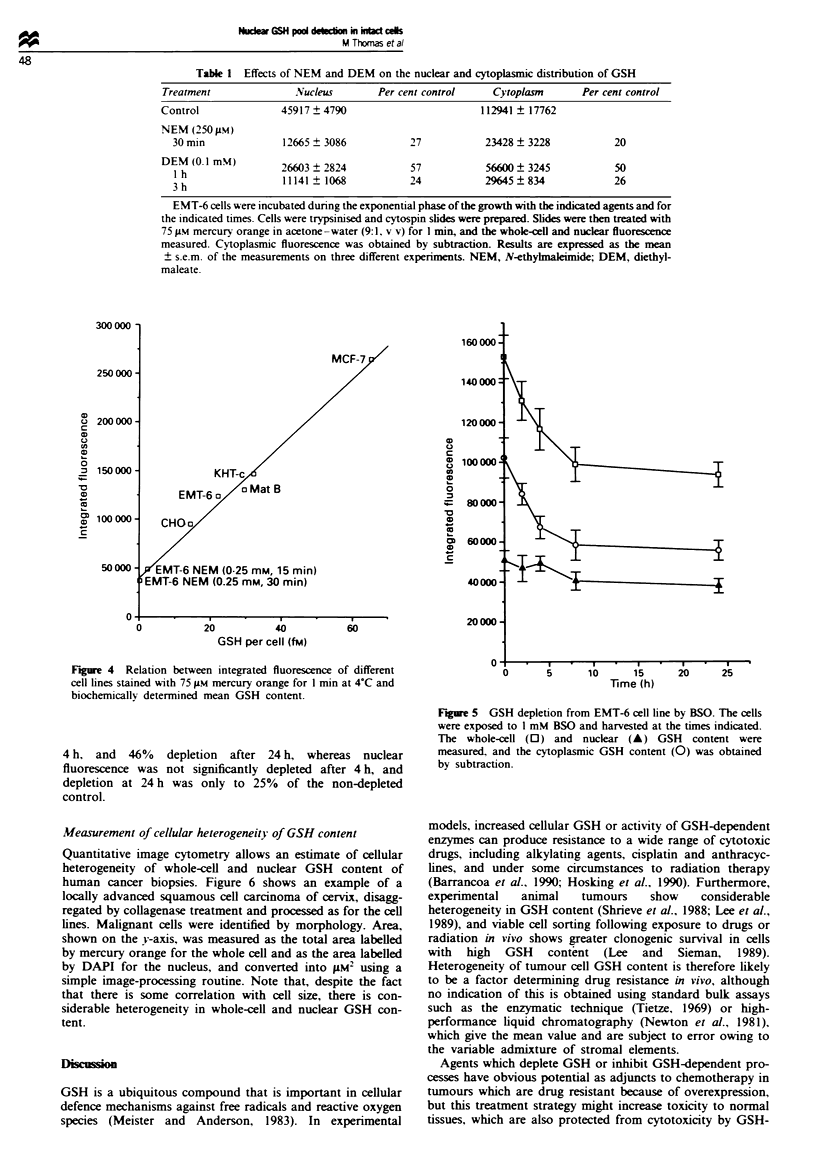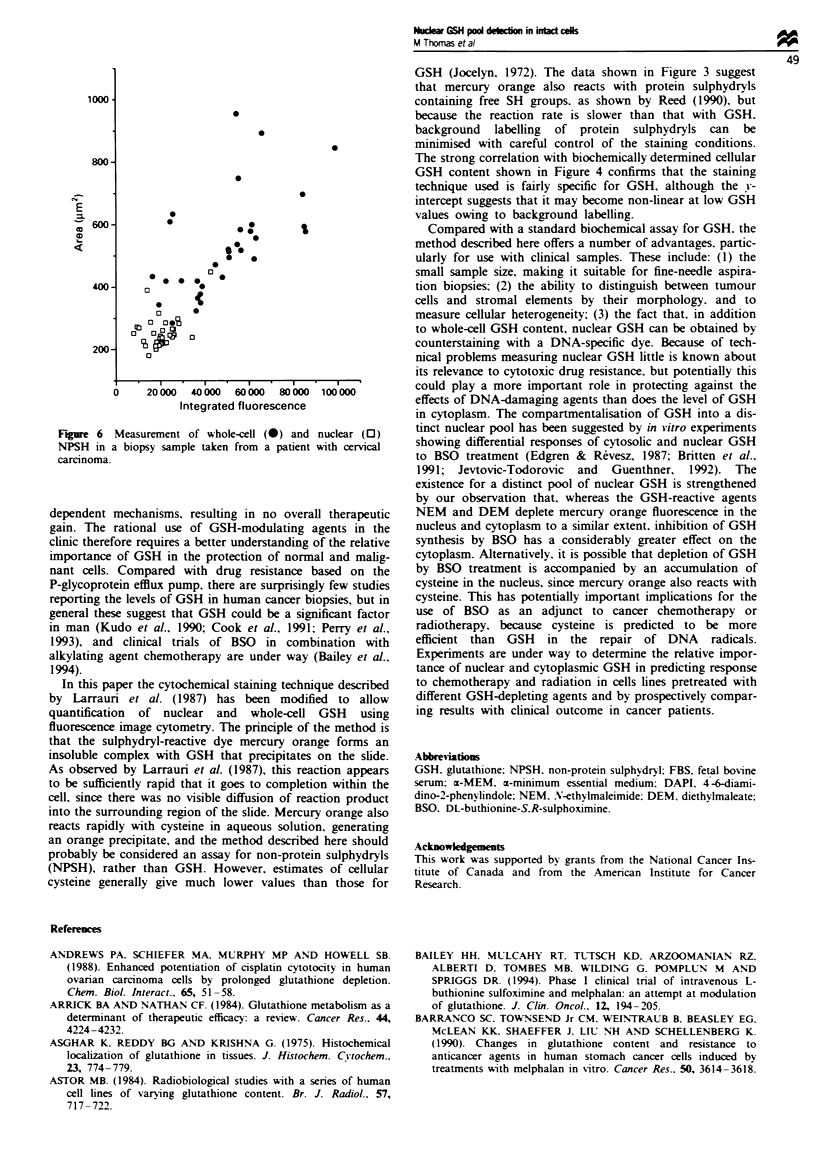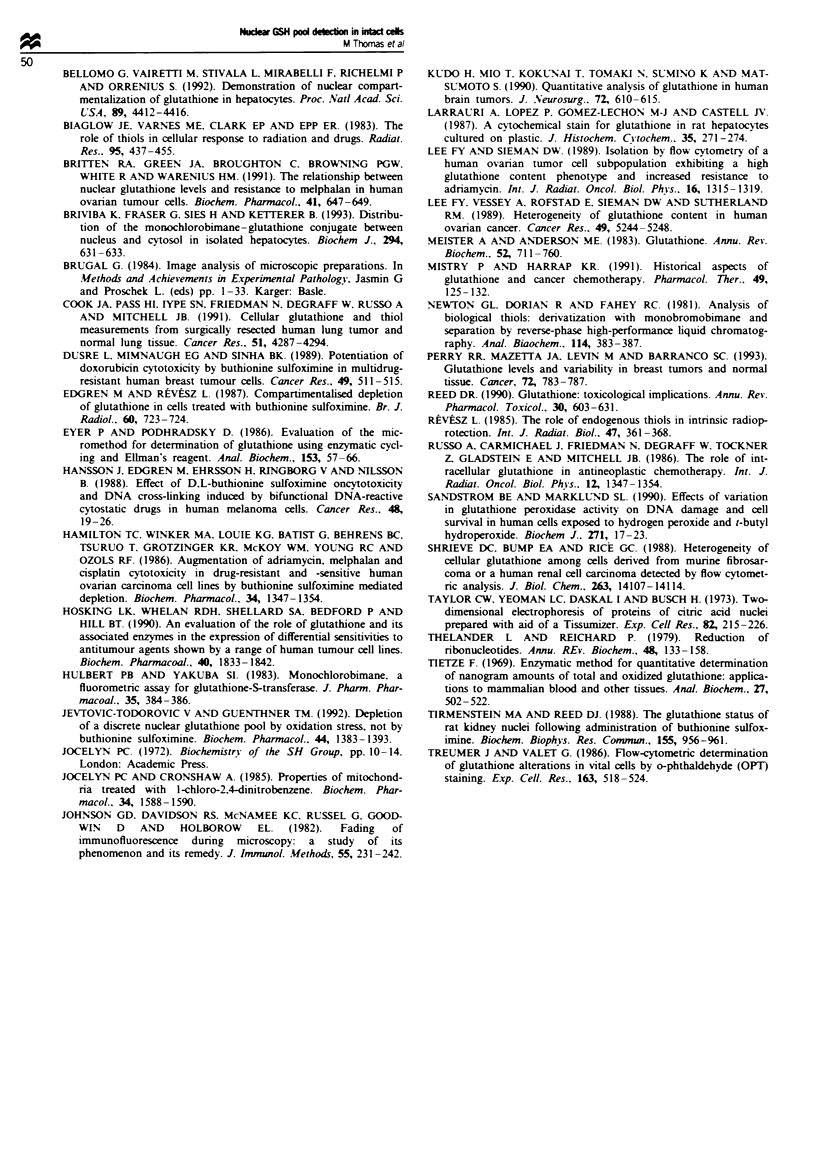# Differential effects of depleting agents on cytoplasmic and nuclear non-protein sulphydryls: a fluorescence image cytometry study.

**DOI:** 10.1038/bjc.1995.275

**Published:** 1995-07

**Authors:** M. Thomas, T. Nicklee, D. W. Hedley

**Affiliations:** Department of Oncologic Pathology, Ontario Cancer Institute/Princess Margaret Hospital, Toronto, Canada.

## Abstract

**Images:**


British Journal of Cancer (1995) 72, 45-50

© 1995 Stockton Press All rights reserved 0007-0920/95 $12.00

### Differential effects of depleting agents on cytoplasmic and nuclear non-protein sulphydryls: a fluorescence image cytometry study

M Thomas*, T Nicklee and DW Hedley

*Cytometry Interface Laboratory, Department of Oncologic Pathology, Ontario Cancer Institute Princess Margaret Hospital, Toronto, Ontario M4X 1K9, Canada.*

**Summary** The intracellular distribution of glutathione (GSH) was measured by a quantitative image cytometry method, using the sulphydryl-reactive agent mercury orange. This readily forms fluorescent adducts with GSH and other non-protein sulphydryls (NPSH), but reacts much more slowly with protein sulphydryls. Under optimum staining conditions mean integrated mercury orange fluorescence per cell was closely correlated with a standard biochemical assay for GSH. Use of the DNA dye DAPI as a counterstain allowed measurement of nuclear NPSH. The mean nuclear-cytoplasmic ratio was $0.57 \pm 0.05$. Isolation of nuclei under aqueous conditions resulted in the loss of approximately 90% of mercury orange fluorescence, compared with nuclear fluorescence from intact cells, suggesting that background labelling of protein sulphydryls or other macromolecules is low. Depletion of GSH with *N*-ethylmaleimide or diethylmaleate decreased mercury orange fluorescence in the nucleus and cytoplasm to a similar extent. In contrast, mercury orange fluorescence in the nucleus was much more resistant to DL-buthionine-*S,R*-sulphoximine (BSO) depletion than that in the cytoplasm. This finding is compatible with a distinct pool of GSH in the nucleus that is comparatively resistant to BSO depletion. Alternatively, the retention of fluorescence in the nucleus following GSH depletion by BSO treatment might be due to accumulation of cysteine. These findings have implications for cancer treatment since the level of NPSH in the nucleus might be a more important determinant of resistance to DNA-damaging agents than that in cytoplasm. The image cytometry method described here is quantitative, allows a measure of tumour cell heterogeneity and can be applied to small biopsy samples obtained by fine-needle aspiration. Thus it appears suitable for prospective clinical studies in cancer patients, and for monitoring the effects of GSH-depleting agents used as adjuncts to cancer chemotherapy or radiotherapy.

**Keywords:** glutathione; drug resistance; mercury orange; image cytometry; buthionine sulphoximine

Glutathione (GSH) plays a key role in the protection of mammalian cells from ionising radiation and anti-cancer drugs (Biaglow *et al*., 1983; Astor, 1984; Révész, 1985; Andrews *et al*., 1988; Hansson *et al*., 1988; Dusre *et al*., 1989). GSH protects the cell (1) by reacting chemically with intracellular targets (mainly DNA), (2) by enzymatically reducing peroxides, (3) by enzymatically detoxifying electrophiles and (4) by maintaining the redox state of cellular thiols. The level of cellular GSH, either constitutive or following biochemical manipulation, has been correlated with the relative susceptibility and resistance of tumour cells to ionising radiation and a variety of anti-tumour agents (Arrich and Nathan, 1984; Hamilton *et al*., 1986; Russo *et al*., 1986; Mistry and Harrap, 1991). In tissue culture GSH is usually considered to be the dominant non-protein sulphydryl (NPSH) involved in drug and radiation resistance. Although cysteine is a more chemically reactive NPSH, it is usually present at much lower concentrations than GSH.

There is good evidence that subcellular GSH pools exist in cytosol and mitochondria, each with different rates of turnover and depletion (Jocelyn and Cronshaw, 1985; Reed, 1990). In order to protect the nuclear structures from damage (Sandstrom and Marklund 1990) and to participate to DNA synthesis (Thelander and Reichard, 1979), GSH must also be present in the nucleoplasm, but little is known about the nuclear GSH content, and the published values for the nuclear-cytoplasmic distribution of GSH vary according to the techniques used (Edgren and Révész, 1987; Tirmenstein and Reed, 1988; Britten *et al*., 1991; Jevtovic-Todorovic and Guenthner, 1992). Taylor *et al*. (1973) found that subcellular fractionation of rat liver by aqueous techniques gave nuclei which contained little or no GSH, while in contrast Tirmensten and Reed (1988) reported that fractionation of rat kidney under non-aqueous conditions gave values for nuclear GSH which were similar to those in the cytoplasm. Other fractionation techniques have provided equivocal results (Tirmenstein and Reed, 1988; Britten *et al*., 1991; Jevtovic-Todorovic and Guenthner, 1992). Recently, Bellomo *et al*. (1992) carried out a study, intended to measure the intracellular distribution of GSH in intact hepatocytes, using the non-fluorescent probe monochlorobimane, which permeates cells and produces a strongly fluorescent bimane-glutathione conjugate under the action of glutathione *S*-transferase (Hulbert and Yakuba, 1983). Although they reported that fluorescence was preferentially localised in the nucleus, with a nuclear-cytoplasmic GSH ratio of about 3:1, Briviba *et al*. (1993) have subsequently shown that a major drawback of monochlorobimane is the ability of the GSH-bimane conjugate to diffuse from the cytoplasm to the nucleus, where it is concentrated.

The sulphydryl-reactive dye mercury orange has been shown to bind much more rapidly to GSH than to protein thiols, and can therefore be used for the histochemical localisation of GSH (Asghar *et al*., 1975). Larrauri *et al*. (1987) have adapted this procedure for measuring GSH in cells cultured on plastic, and shown that the reaction product between GSH and mercury orange precipitated inside the cell with no appreciable diffusion into the supernatant. We have now modified this method by using fluorescence image cytometry to allow quantification of GSH in the whole cell and nucleus, and have found evidence for the existence of a distinct pool of GSH in the cell nucleus which is resistant to depletion by buthionine sulphoximine, an inhibitor of *de novo* synthesis.

### Materials and methods

### Cell lines and sample preparation

All cell lines used were grown in 25 $cm^3$ Corning flasks in a humidified 5% carbon dioxide/air incubator at 37°C. Mouse mammary carcinosarcoma EMT-6 cells were grown as a

Correspondence: DW Hedley
*Present address: Department of Cell Biology, Baylor College of Medicine, One Baylor Plaza, Houston, Texas 77030, USA
Received 18 November 1994; revised 14 February 1995; accepted 16 February 1995

monolayer culture in α-minimum essential medium (α-MEM) supplemented with 5% fetal bovine serum (FBS) (PA Biologicals, Sydney, Australia). Chinese hamster ovary cells were maintained as spinner cultures in α-MEM supplemented with 5% FBS. The human breast cancer cell line MCF-7 and the mouse lung fibrosarcoma cell line KHT-c were grown in α-MEM supplemented with 10% FBS and the rat mammary carcinoma Mat B in α-MEM supplemented with 10% FBS, 1% L-glutamine 200 mM (Gibco BRL, Grand Island, NY, USA) and 1% hypoxanthine (Gibco). Cells were used during the exponential phase of growth.

Cells were removed from the monolayer using 0.05% trypsin and 0.53 mM EDTA (Gibco) for 5 min at 37°C and resuspended in α-MEM supplemented with 5% or 10% of FBS at a final concentration of $1 \times 10^5$ cells $ml^{-1}$. Slide preparations were prepared using a cytocentrifuge (Cytospin 3, Shandon Elliot, Astmoor, UK), a total of 7500 cells being spread on each slide. Slides were air dried for at least 3 h to evaporate all intracellular water, wrapped and kept at −70°C. Before mercury orange staining the slides were thawed for 1 h and unwrapped.

*Staining with mercury orange*

Mercury orange [1(4-chloromercuryphenyl-azo-2-naphthol); Sigma, St. Louis, MO, USA)] was first dissolved in acetone and later brought to the concentration required in 9:1 (v/v) acetone–water and kept at 4°C. The staining solution was added to slides in Coplin jars and allowed to stain for different times at 4°C. The staining solution was then removed and the slides were thoroughly washed with acetone–water (9:1, v/v) for 5 min to remove excess staining solution and then washed twice for 5 min with distilled water and allowed to air dry for 10 min.

Slides were counterstained with the DNA-specific dye 4,6-diamidino-2-phenylindole (DAPI, Sigma) at 1 μg $ml^{-1}$ for 5 min at 4°C, then rinsed twice for 5 min in phosphate-buffered salaine (PBS) to remove background fluorescence. Finally, the slides were mounted with a solution of 9:1 glycerol–PBS containing 0.23% 1,4-diazabicyclo [2.2.2.] octane (Sigma), an antifading compound (Johnson *et al.*, 1982). Slides were immediately analysed or kept at 4°C for less than 1 week before being analysed. Autofluorescence of the cells was estimated by treating slides as mentioned above but omitting the mercury orange from the staining solution.

*Quantitative fluorescence microscopy*

This was done using a SAMBA 4000 image cytometer (IPI, Chantilly, VA, USA). The system consists of a reflected fluorescence microscope (BX50, Olympus) fitted with a 100 W mercury arc lamp, connected to an intensified charge-coupled device (CCD) camera (XC 77, Hamamastu, Japan), a preprocessor (Matrox Mip), an image analysis processor and a host computer (Victor 386). The hardware and software packages of the system have already been described elsewhere (Brugal, 1984).

Each fluorescent image was obtained through a × 40, NA 0.75 dry objective and digitised into a 512 × 480 image frame onto 256 levels. For mercury orange fluorescence, cells were excited at 530–560 nm and the images were obtained by using a 570 nm dichroic mirror and a 590 nm long-pass barrier filter. For DAPI fluorescence, the excitation wavelength was 360–370 nm and images were acquired by using a 400 nm dichroic mirror and a 420 nm long-pass barrier filter.

Image analysis involved acquisition of the background signal of the cell preparation in order to correct for possible non-specific fluorescence for each fluorochrome, and the interactive adjustment of the thresholds for cell selection and segmentation. Image cytometry was carried out on 30–40 cells per slide as follows. The cells were first examined with ultraviolet light and a digitised image of the nucleus as defined by DAPI fluorescence recorded. The same field was then examined with green light and a digitised image of mercury orange fluorescence recorded for the whole cell. Using an image analysis program, the integrated fluorescence was derived for the entire cell using mercury orange and for the nuclear area defined by DAPI staining, and the cytoplasmic GSH content obtained by subtraction.

*Biochemical assay of cellular glutathione*

To determine the specificity and linearity of the fluorescence image cytometry method a comparison was made with a standard biochemical assay for GSH, using preparations made from the same population of cells. This was based on the glutathione reductase recycling method originally described by Tietze (1969). The principle is the reduction by GSH of the disulphide 5,5′-dithio-2-nitrobenzoic acid (DTNB), yielding 2-nitro-5-thiobenzoic acid, which absorbs at 412 nm. In the process GSH is oxidised, and the reaction is maintained by adding the enzyme glutathione reductase and the electron donor NAPDH, so that the concentration of GSH is rate limiting.

The cells were counted in a haemocytometer and $1 \times 10^6$ viable cells were resuspended in an Eppendorf tube in 0.5 ml of cold 0.6% sulphosalicyclic acid (Sigma) made up in distilled water. They were left on ice for 1 h, then spun down at 14 000 r.p.m. for 15 min at 4°C. The supernatant was transferred to a new Eppendorf tube and stored at −20°C. The assay was a modification of that described by Eyer and Podhradsky (1986). A 50 μl sample was diluted to 1 ml in PBS containing 60 μg of DTNB (Sigma), 200 μg of NADPH (Sigma), and 1 unit of glutathione reductase (Sigma). The reaction rate was monitored using a spectrophotometer (Cary, Varian Instruments), measuring absorption at 412 nm over time. The GSH content of the sample was determined by comparing the reaction rate with that obtained using a series of known GSH concentrations.

*Modification of cellular glutathione content*

In addition to comparing values for the image cytometry and biochemical assays obtained for untreated cells, we investigated the effects of a variety of agents which deplete GSH by different mechanisms. *N*-Ethylmaleimide (NEM, Sigma) chemically blocks -SH groups present in the cells, and was used at a concentration of 250 μM for 30 min. Diethylmaleate (DEM, Sigma) is conjugated to GSH under the action of GSH transferases, and is therefore more specific than NEM. It was added to the monolayers at a final concentration of 100 μM. Buthionine sulphoximine (BSO) is a potent and specific inhibitor of γ-glutamylcysteine synthetase, the rate-limiting enzyme catalysing the first step of GSH biosynthesis, and was used at a concentration of 1 mM for various times.

### Results

*Measurement of cellular GSH using mercury orange*

Mercury orange staining produced a bright orange–red fluorescence under excitation from the strong green emission of the mercury arc lamp (Figure 1a), and the nuclear indicator dye DAPI revealed the localisation of the nucleus (Figure 1b). A range of conditions was investigated in order to ensure that staining of GSH was saturated while minimising background labelling of protein sulphydryls. The optimum stain concentration was found to be 75 μM, made up in 90% acetone, 10% water and used at 4°C, as described by Larrauri *et al.*, (1987) (Figure 2). The rate of reaction with GSH and the specificity of staining were investigated using cells which had been variably depleted of GSH by treatment with BSO. As shown in Figure 3, GSH labelling with mercury orange achieved saturation within 30 s, whereas longer staining times resulted in increased background staining. Approximately 90% of this background was eliminated by pretreatment of slides with 1 mM mercuric chloride, which causes profound loss of all available reduced sulphydryls (Treumer and Valet, 1986), indicating that it was substan-

**Figure 1** Intracellular localisation of GSH in EMT-6 cells. (**a**) Fluorescent image of the intracellular distribution of mercury orange-GSH adducts. (**b**) Double exposure showing dual staining with mercury orange and the DNA dye DAPI (blue fluorescent; × 340).

**Figure 2** Relationship between mercury orange concentration and the amount of final reaction product expressed as integrated fluorescence per cell.

**Figure 3** Effect of incubation time on the generation of fluorescence in control cells (□) and cells treated with 1 mM BSO for 24 h (●) stained with 75 μM mercury orange for 1 min at 4°C. Subtraction of these curves gives the time course for specific labelling of GSH (inset).

tially due to binding of mercury orange to protein thiols. A comparison of biochemically determined GSH content and mean integrated mercury orange fluorescence obtained for the same cell population is shown in Figure 4. There was a strong, linear correlation between the two measurements over a wide range of GSH values. The *y*-intercept, which represents background mercury orange fluorescence when biochemically determined GSH = 0, was comparatively low. These results indicate that the image cytometry method is capable of giving reliable estimates of cellular GSH content.

*Measurement of nuclear GSH*

The nuclear-cytoplasmic ratio for mercury orange labelling of non-GSH-depleted, intact EMT-6 cells ranged from 0.4 to 0.68 (mean = 0.57 ± 0.05). Nuclei were isolated from suspensions of EMT-6 cells by hypotonic lysis. Approximately $5 \times 10^5$ cells were pelleted and 1 ml of hypotonic solution (584 mg $l^{-1}$ sodium chloride, 1000 mg $l^{-1}$ sodium citrate and 0.3 ml $l^{-1}$ Nonidet P40) added for 5 min at room temperature. Cells were then centrifuged at 1000 r.p.m. for 5 min at 5°C, and an aliquot was stained with trypan blue to confirm that the preparation was free from contamination with intact cells. Slides were prepared and stained as for whole EMT-6 cells. The integrated fluorescence recorded for isolated nuclei was only 10.5% of the integrated fluorescence for nuclei from whole cells. This suggests that the bulk of nuclear fluorescence is derived from NPSH, rather than from proteins or other macromolecules. The effects of GSH-depleting agents on nuclear GSH were then investigated under a range of conditions. As shown in Table I, depletion of GSH by alkylation with NEM or by enzymatic conjugation to DEM markedly decreased GSH content measured by mercury orange fluorescence, and for both agents the nuclear and cytoplasmic GSH contents were depleted to a similar extent. In contrast, incubation with BSO, which is a specific inhibitor of *de novo* GSH synthesis and thus reduces the level of GSH in a time-dependent manner, produced a much greater rate of GSH depletion in cytoplasm than in the nucleus (Figure 5). Exposure to 1 mM BSO resulted in 34% depletion of the cytoplasmic mercury orange fluorescence at

**Table 1** Effects of NEM and DEM on the nuclear and cytoplasmic distribution of GSH

| *Treatment* | *Nucleus* | *Per cent control* | *Cytoplasm* | *Per cent control* |
|---|---|---|---|---|
| Control | 45917 ± 4790 | | 112941 ± 17762 | |
| NEM (250 μM) | | | | |
| 30 min | 12665 ± 3086 | 27 | 23428 ± 3228 | 20 |
| DEM (0.1 mM) | | | | |
| 1 h | 26603 ± 2824 | 57 | 56600 ± 3245 | 50 |
| 3 h | 11141 ± 1068 | 24 | 29645 ± 834 | 26 |

EMT-6 cells were incubated during the exponential phase of the growth with the indicated agents and for the indicated times. Cells were trypsinised and cytospin slides were prepared. Slides were then treated with 75 μM mercury orange in acetone-water (9:1, v v) for 1 min, and the whole-cell and nuclear fluorescence measured. Cytoplasmic fluorescence was obtained by subtraction. Results are expressed as the mean ± s.e.m. of the measurements on three different experiments. NEM, *N*-ethylmaleimide; DEM, diethylmaleate.

**Figure 4** Relation between integrated fluorescence of different cell lines stained with 75 μM mercury orange for 1 min at 4°C and biochemically determined mean GSH content.

**Figure 5** GSH depletion from EMT-6 cell line by BSO. The cells were exposed to 1 mM BSO and harvested at the times indicated. The whole-cell (□) and nuclear (▲) GSH content were measured, and the cytoplasmic GSH content (O) was obtained by subtraction.

4 h, and 46% depletion after 24 h, whereas nuclear fluorescence was not significantly depleted after 4 h, and depletion at 24 h was only to 25% of the non-depleted control.

### *Measurement of cellular heterogeneity of GSH content*

Quantitative image cytometry allows an estimate of cellular heterogeneity of whole-cell and nuclear GSH content of human cancer biopsies. Figure 6 shows an example of a locally advanced squamous cell carcinoma of cervix, disaggregated by collagenase treatment and processed as for the cell lines. Malignant cells were identified by morphology. Area, shown on the *y*-axis, was measured as the total area labelled by mercury orange for the whole cell and as the area labelled by DAPI for the nucleus, and converted into $\mu M^2$ using a simple image-processing routine. Note that, despite the fact that there is some correlation with cell size, there is considerable heterogeneity in whole-cell and nuclear GSH content.

### Discussion

GSH is a ubiquitous compound that is important in cellular defence mechanisms against free radicals and reactive oxygen species (Meister and Anderson, 1983). In experimental models, increased cellular GSH or activity of GSH-dependent enzymes can produce resistance to a wide range of cytotoxic drugs, including alkylating agents, cisplatin and anthracyclines, and under some circumstances to radiation therapy (Barrancoa *et al.*, 1990; Hosking *et al.*, 1990). Furthermore, experimental animal tumours show considerable heterogeneity in GSH content (Shrieve *et al.*, 1988; Lee *et al.*, 1989), and viable cell sorting following exposure to drugs or radiation *in vivo* shows greater clonogenic survival in cells with high GSH content (Lee and Sieman, 1989). Heterogeneity of tumour cell GSH content is therefore likely to be a factor determining drug resistance *in vivo*, although no indication of this is obtained using standard bulk assays such as the enzymatic technique (Tietze, 1969) or high-performance liquid chromatography (Newton *et al.*, 1981), which give the mean value and are subject to error owing to the variable admixture of stromal elements.

Agents which deplete GSH or inhibit GSH-dependent processes have obvious potential as adjuncts to chemotherapy in tumours which are drug resistant because of overexpression, but this treatment strategy might increase toxicity to normal tissues, which are also protected from cytotoxicity by GSH-

**Figure 6** Measurement of whole-cell (●) and nuclear (□) NPSH in a biopsy sample taken from a patient with cervical carcinoma.

dependent mechanisms, resulting in no overall therapeutic gain. The rational use of GSH-modulating agents in the clinic therefore requires a better understanding of the relative importance of GSH in the protection of normal and malignant cells. Compared with drug resistance based on the P-glycoprotein efflux pump, there are surprisingly few studies reporting the levels of GSH in human cancer biopsies, but in general these suggest that GSH could be a significant factor in man (Kudo *et al.*, 1990; Cook *et al.*, 1991; Perry *et al.*, 1993), and clinical trials of BSO in combination with alkylating agent chemotherapy are under way (Bailey *et al.*, 1994).

In this paper the cytochemical staining technique described by Larrauri *et al.* (1987) has been modified to allow quantification of nuclear and whole-cell GSH using fluorescence image cytometry. The principle of the method is that the sulphydryl-reactive dye mercury orange forms an insoluble complex with GSH that precipitates on the slide. As observed by Larrauri *et al.* (1987), this reaction appears to be sufficiently rapid that it goes to completion within the cell, since there was no visible diffusion of reaction product into the surrounding region of the slide. Mercury orange also reacts rapidly with cysteine in aqueous solution, generating an orange precipitate, and the method described here should probably be considered an assay for non-protein sulphydryls (NPSH), rather than GSH. However, estimates of cellular cysteine generally give much lower values than those for GSH (Jocelyn, 1972). The data shown in Figure 3 suggest that mercury orange also reacts with protein sulphydryls containing free SH groups, as shown by Reed (1990), but because the reaction rate is slower than that with GSH, background labelling of protein sulphydryls can be minimised with careful control of the staining conditions. The strong correlation with biochemically determined cellular GSH content shown in Figure 4 confirms that the staining technique used is fairly specific for GSH, although the *y*-intercept suggests that it may become non-linear at low GSH values owing to background labelling.

Compared with a standard biochemical assay for GSH, the method described here offers a number of advantages, particularly for use with clinical samples. These include: (1) the small sample size, making it suitable for fine-needle aspiration biopsies; (2) the ability to distinguish between tumour cells and stromal elements by their morphology, and to measure cellular heterogeneity; (3) the fact that, in addition to whole-cell GSH content, nuclear GSH can be obtained by counterstaining with a DNA-specific dye. Because of technical problems measuring nuclear GSH little is known about its relevance to cytotoxic drug resistance, but potentially this could play a more important role in protecting against the effects of DNA-damaging agents than does the level of GSH in cytoplasm. The compartmentalisation of GSH into a distinct nuclear pool has been suggested by *in vitro* experiments showing differential responses of cytosolic and nuclear GSH to BSO treatment (Edgren & Révesz, 1987; Britten *et al.*, 1991; Jevtovic-Todorovic and Guenthner, 1992). The existence for a distinct pool of nuclear GSH is strengthened by our observation that, whereas the GSH-reactive agents NEM and DEM deplete mercury orange fluorescence in the nucleus and cytoplasm to a similar extent, inhibition of GSH synthesis by BSO has a considerably greater effect on the cytoplasm. Alternatively, it is possible that depletion of GSH by BSO treatment is accompanied by an accumulation of cysteine in the nucleus, since mercury orange also reacts with cysteine. This has potentially important implications for the use of BSO as an adjunct to cancer chemotherapy or radiotherapy, because cysteine is predicted to be more efficient than GSH in the repair of DNA radicals. Experiments are under way to determine the relative importance of nuclear and cytoplasmic GSH in predicting response to chemotherapy and radiation in cells lines pretreated with different GSH-depleting agents and by prospectively comparing results with clinical outcome in cancer patients.

### Abbreviations

GSH, glutathione; NPSH, non-protein sulphydryl; FBS, fetal bovine serum; α-MEM, α-minimum essential medium; DAPI, 4′-6-diamidino-2-phenylindole; NEM, *N*-ethylmaleimide; DEM, diethylmaleate; BSO, DL-buthionine-*S,R*-sulphoximine.

### Acknowledgements

This work was supported by grants from the National Cancer Institute of Canada and from the American Institute for Cancer Research.

### References

ANDREWS PA, SCHIEFER MA, MURPHY MP AND HOWELL SB. (1988). Enhanced potentiation of cisplatin cytotocity in human ovarian carcinoma cells by prolonged glutathione depletion. *Chem. Biol. Interact.*, **65**, 51–58.

ARRICK BA AND NATHAN CF. (1984). Glutathione metabolism as a determinant of therapeutic efficacy: a review. *Cancer Res.*, **44**, 4224–4232.

ASGHAR K, REDDY BG AND KRISHNA G. (1975). Histochemical localization of glutathione in tissues. *J. Histochem. Cytochem.*, **23**, 774–779.

ASTOR MB. (1984). Radiobiological studies with a series of human cell lines of varying glutathione content. *Br. J. Radiol.*, **57**, 717–722.

BAILEY HH, MULCAHY RT, TUTSCH KD, ARZOOMANIAN RZ, ALBERTI D, TOMBES MB, WILDING G, POMPLUN M AND SPRIGGS DR. (1994). Phase I clinical trial of intravenous L-buthionine sulfoximine and melphalan: an attempt at modulation of glutathione. *J. Clin. Oncol.*, **12**, 194–205.

BARRANCO SC, TOWNSEND Jr CM, WEINTRAUB B, BEASLEY EG, McLEAN KK, SHAEFFER J, LIU NH AND SCHELLENBERG K. (1990). Changes in glutathione content and resistance to anticancer agents in human stomach cancer cells induced by treatments with melphalan in vitro. *Cancer Res.*, **50**, 3614–3618.

BELLOMO G, VAIRETTI M, STIVALA L, MIRABELLI F, RICHELMI P AND ORRENIUS S. (1992). Demonstration of nuclear compartmentalization of glutathione in hepatocytes. *Proc. Natl Acad. Sci. USA*, **89**, 4412–4416.

BIAGLOW JE, VARNES ME, CLARK EP AND EPP ER. (1983). The role of thiols in cellular response to radiation and drugs. *Radiat. Res.*, **95**, 437–455.

BRITTEN RA, GREEN JA, BROUGHTON C, BROWNING PGW, WHITE R AND WARENIUS HM. (1991). The relationship between nuclear glutathione levels and resistance to melphalan in human ovarian tumour cells. *Biochem. Pharmacol.*, **41**, 647–649.

BRIVIBA K, FRASER G, SIES H AND KETTERER B. (1993). Distribution of the monochlorobimane-glutathione conjugate between nucleus and cytosol in isolated hepatocytes. *Biochem J.*, **294**, 631–633.

BRUGAL G. (1984). Image analysis of microscopic preparations. In *Methods and Achievements in Experimental Pathology*. Jasmin G and Proschek L. (eds) pp. 1–33. Karger: Basle.

COOK JA, PASS HI, IYPE SN, FRIEDMAN N, DEGRAFF W, RUSSO A AND MITCHELL JB. (1991). Cellular glutathione and thiol measurements from surgically resected human lung tumor and normal lung tissue. *Cancer Res.*, **51**, 4287–4294.

DUSRE L, MIMNAUGH EG AND SINHA BK. (1989). Potentiation of doxorubicin cytotoxicity by buthionine sulfoximine in multidrug-resistant human breast tumour cells. *Cancer Res.*, **49**, 511–515.

EDGREN M AND RÉVÉSZ L. (1987). Compartimentalised depletion of glutathione in cells treated with buthionine sulfoximine. *Br. J. Radiol.*, **60**, 723–724.

EYER P AND PODHRADSKY D. (1986). Evaluation of the micromethod for determination of glutathione using enzymatic cycling and Ellman's reagent. *Anal. Biochem.*, **153**, 57–66.

HANSSON J, EDGREN M, EHRSSON H, RINGBORG V AND NILSSON B. (1988). Effect of D,L-buthionine sulfoximine oncytotoxicity and DNA cross-linking induced by bifunctional DNA-reactive cytostatic drugs in human melanoma cells. *Cancer Res.*, **48**, 19–26.

HAMILTON TC, WINKER MA, LOUIE KG, BATIST G, BEHRENS BC, TSURUO T, GROTZINGER KR, McKOY WM, YOUNG RC AND OZOLS RF. (1986). Augmentation of adriamycin, melphalan and cisplatin cytotoxicity in drug-resistant and -sensitive human ovarian carcinoma cell lines by buthionine sulfoximine mediated depletion. *Biochem. Pharmacol.*, **34**, 1347–1354.

HOSKING LK, WHELAN RDH, SHELLARD SA, BEDFORD P AND HILL BT. (1990). An evaluation of the role of glutathione and its associated enzymes in the expression of differential sensitivities to antitumour agents shown by a range of human tumour cell lines. *Biochem. Pharmacoal.*, **40**, 1833–1842.

HULBERT PB AND YAKUBA SI. (1983). Monochlorobimane, a fluorometric assay for glutathione-S-transferase. *J. Pharm. Pharmacoal.*, **35**, 384–386.

JEVTOVIC-TODOROVIC V AND GUENTHNER TM. (1992). Depletion of a discrete nuclear glutathione pool by oxidation stress, not by buthionine sulfoximine. *Biochem. Pharmacol.*, **44**, 1383–1393.

JOCELYN PC. (1972). *Biochemistry of the SH Group*, pp. 10–14. London: Academic Press.

JOCELYN PC AND CRONSHAW A. (1985). Properties of mitochondria treated with 1-chloro-2,4-dinitrobenzene. *Biochem. Pharmacol.*, **34**, 1588–1590.

JOHNSON GD, DAVIDSON RS, McNAMEE KC, RUSSEL G, GOODWIN D AND HOLBOROW EL. (1982). Fading of immunofluorescence during microscopy: a study of its phenomenon and its remedy. *J. Immunol. Methods*, **55**, 231–242.

KUDO H, MIO T, KOKUNAI T, TOMAKI N, SUMINO K AND MATSUMOTO S. (1990). Quantitative analysis of glutathione in human brain tumors. *J. Neurosurg.*, **72**, 610–615.

LARRAURI A, LOPEZ P, GOMEZ-LECHON M-J AND CASTELL JV. (1987). A cytochemical stain for glutathione in rat hepatocytes cultured on plastic. *J. Histochem. Cytochem.*, **35**, 271–274.

LEE FY AND SIEMAN DW. (1989). Isolation by flow cytometry of a human ovarian tumor cell subpopulation exhibiting a high glutathione content phenotype and increased resistance to adriamycin. *Int. J. Radiat. Oncol. Biol. Phys.*, **16**, 1315–1319.

LEE FY, VESSEY A, ROFSTAD E, SIEMAN DW AND SUTHERLAND RM. (1989). Heterogeneity of glutathione content in human ovarian cancer. *Cancer Res.*, **49**, 5244–5248.

MEISTER A AND ANDERSON ME. (1983). Glutathione. *Annu. Rev. Biochem.*, **52**, 711–760.

MISTRY P AND HARRAP KR. (1991). Historical aspects of glutathione and cancer chemotherapy. *Pharmacol. Ther.*, **49**, 125–132.

NEWTON GL, DORIAN R AND FAHEY RC. (1981). Analysis of biological thiols: derivatization with monobromobimane and separation by reverse-phase high-performance liquid chromatography. *Anal. Biaochem.*, **114**, 383–387.

PERRY RR, MAZETTA JA, LEVIN M AND BARRANCO SC. (1993). Glutathione levels and variability in breast tumors and normal tissue. *Cancer*, **72**, 783–787.

REED DR. (1990). Glutathione: toxicological implications. *Annu. Rev. Pharmacol. Toxicol.*, **30**, 603–631.

RÉVÉSZ L. (1985). The role of endogenous thiols in intrinsic radioprotection. *Int. J. Radiat. Biol.*, **47**, 361–368.

RUSSO A, CARMICHAEL J, FRIEDMAN N, DEGRAFF W, TOCKNER Z, GLADSTEIN E AND MITCHELL JB. (1986). The role of intracellular glutathione in antineoplastic chemotherapy. *Int. J. Radiat. Oncol. Biol. Phys.*, **12**, 1347–1354.

SANDSTROM BE AND MARKLUND SL. (1990). Effects of variation in glutathione peroxidase activity on DNA damage and cell survival in human cells exposed to hydrogen peroxide and *t*-butyl hydroperoxide. *Biochem J.*, **271**, 17–23.

SHRIEVE DC, BUMP EA AND RICE GC. (1988). Heterogeneity of cellular glutathione among cells derived from murine fibrosarcoma or a human renal cell carcinoma detected by flow cytometric analysis. *J. Biol. Chem.*, **263**, 14107–14114.

TAYLOR CW, YEOMAN LC, DASKAL I AND BUSCH H. (1973). Two-dimensional electrophoresis of proteins of citric acid nuclei prepared with aid of a Tissumizer. *Exp. Cell Res.*, **82**, 215–226.

THELANDER L AND REICHARD P. (1979). Reduction of ribonucleotides. *Annu. REv. Biochem.*, **48**, 133–158.

TIETZE F. (1969). Enzymatic method for quantitative determination of nanogram amounts of total and oxidized glutathione: applications to mammalian blood and other tissues. *Anal. Biochem.*, **27**, 502–522.

TIRMENSTEIN MA AND REED DJ. (1988). The glutathione status of rat kidney nuclei following administration of buthionine sulfoximine. *Biochem. Biophys. Res. Commun.*, **155**, 956–961.

TREUMER J AND VALET G. (1986). Flow-cytometric determination of glutathione alterations in vital cells by o-phthaldehyde (OPT) staining. *Exp. Cell. Res.*, **163**, 518–524.